# Meso-Scale Modifications in Additively Manufactured Zirconia: Topographical Design and Its Influence on Cell–Material Interactions

**DOI:** 10.3390/bioengineering13050498

**Published:** 2026-04-24

**Authors:** Sebastian Hetzler, Stefan Rues, Andreas Zenthöfer, Peter Rammelsberg, Reinald Kühle, Christopher J. Lux, Ralf Erber, Christoph J. Roser

**Affiliations:** 1 Department of Prosthodontics, Medical Faculty, Heidelberg University, Im Neuenheimer Feld 400, 69120 Heidelberg, Germany; 2 Deparment of Craniomaxillofacial Surgery, Medical Facutly, Heidelberg University, Im Neuenheimer Feld 400, 69120 Heidelberg, Germany; 3 Department of Orthodontics and Dentofacial Orthopedics, Medical Faculty, Heidelberg University, Im Neuenheimer Feld 400, 69120 Heidelberg, Germany

**Keywords:** regenerative medicine, personalized dentistry, additive manufacturing, digital light processing (DLP), zirconia, 3Y-TZP, 5Y-PSZ, meso-scale surface topography, osteoblast adhesion, osteogenic differentiation, dental implants, cell–material interaction

## Abstract

Additive manufacturing enables the fabrication of patient-specific zirconia devices with integrated surface features; however, the biological effects of meso-scale topographies remain insufficiently understood. This in vitro study evaluated the influence of defined meso-scale surface modifications on osteoblast behavior using Digital Light Processing (DLP)-fabricated 3Y tetragonal zirconia polycrystal (3Y-TZP) and 5Y partially stabilized zirconia (5Y-PSZ). Planar control specimens and surfaces incorporating regularly distributed columnar structures (height: 100 µm; width: 40 µm; center-to-center spacing: 80, 120, and 160 µm; Mod-80, Mod-120, Mod-160) were fabricated and characterized after sintering. Cytotoxicity was assessed by elution testing and showed cell viability >98% for all groups. Osteoblast adhesion and proliferation (hFOB 1.19) were quantified using metabolic assays. Meso-scale modifications significantly increased early cell adhesion compared to planar controls (*p* < 0.05), with the strongest effect observed for Mod-160. No significant differences in proliferation rates were detected between groups (*p* > 0.05). Osteogenic differentiation was evaluated by RT-qPCR (RUNX2, ALPL, COL1A1, BGLAP), revealing material- and geometry-dependent responses. On 3Y-TZP, meso-scale structures, particularly Mod-160, were associated with sustained upregulation of BGLAP, whereas 5Y-PSZ exhibited less pronounced effects. Within the limitations of this in vitro study, meso-scale surface structuring of additively manufactured zirconia enhances early osteoblast adhesion without affecting proliferation and may influence osteogenic differentiation in a material-dependent manner.

## 1. Introduction

Zirconia (ZrO_2_), particularly tetragonal zirconia polycrystal doped with 3 mol% yttria (3Y-TZP), has emerged as a clinically relevant biomaterial in dentistry [1] and oral implantology [2] owing to its high fracture toughness, wear resistance, corrosion stability, and favorable biocompatibility. In addition to its mechanical reliability, zirconia demonstrates reduced bacterial adhesion [3], and a diminished inflammatory response and bone resorption rate [4] when compared with titanium implant materials.

Clinical studies have shown that zirconia implants exhibit mid-term survival and success rates comparable to titanium systems, provided that appropriate implant design and surface characteristics are applied [5,6,7]. A recent systematic review and meta-analysis (25 studies, 4017 implants) reported a 10-year cumulative survival rate of 95.1% for zirconia implants, with mean marginal bone loss ranging from 0.63 to 2.06 mm over observation periods of up to 132 months [5]. Across studies, success rates for zirconia implants ranged from 57.5% to 93.3%, comparable to the 57.1–100% range reported for titanium implants [6]. These data support the suitability of zirconia as a long-term implant material, while also highlighting that surface characteristics remain a key determinant of clinical outcome.

Conventionally, zirconia implants and prosthetic restorations are fabricated using subtractive CAD/CAM milling. While this manufacturing approach provides good dimensional accuracy and material homogeneity, it is associated with several limitations. Subtractive manufacturing leads to significant material waste, restricts geometric complexity, and frequently necessitates post-fabrication surface modifications such as sandblasting or acid etching to enhance osseointegration [8,9,10]. In contrast, DLP-printed 3Y-TZP zirconia achieves sintered relative densities of up to 99.71 ± 0.15% of theoretical density, with linear shrinkage ratios of approximately 1.28–1.34 depending on build orientation [11,12]. Dimensional accuracy of DLP-sintered zirconia prostheses has been reported at root mean square deviations of 48 ± 9 µm for intaglio surfaces, values comparable to milled glass–ceramic restorations [12]. These figures underscore that DLP-based additive manufacturing is not only geometrically versatile but is also capable of achieving the dimensional fidelity required for clinically relevant dental applications.

Additive manufacturing (AM) of zirconia has therefore gained increasing attention as an alternative fabrication strategy for dental and maxillofacial applications. Importantly, AM offers an unprecedented degree of geometric freedom, enabling the fabrication of undercuts, internal cavities, graded porosities, and patient-specific designs that are difficult or impossible to realize with subtractive techniques [11,12].

This design freedom is particularly relevant for individualized root-analogue implants (RAIs) aiming to enhance the congruence between implant and extraction socket to optimize peri-implant stress distribution and potentially reduce surgical trauma and early marginal bone loss. Clinical and preclinical studies have suggested that RAIs may facilitate immediate implantation with high primary stability and favorable stress distribution [13]. While RAIs have traditionally been fabricated by subtractive milling [14], additive manufacturing offers clear advantages for reproducing complex root geometries, including concavities and undercuts, with a high degree of anatomical fidelity [15,16]. Following the tendency of individualizing patient solutions, the additive manufacturing process has even revived the historic concept of the subperiosteal implant [17]. Utilizing the same concept for a different indication, additive-manufactured scaffolds prove effective for preprosthetic bone augmentation [18].

Beyond overall implant geometry, surface characteristics are recognized as key determinants of implant osseointegration. Osteoblast adhesion, proliferation, and differentiation are strongly influenced by surface topography, roughness, and microstructural features. Micro- and nanoscale surface modifications have been shown to modulate protein adsorption, focal adhesion formation, cytoskeletal organization, and osteogenic gene expression [19,20,21,22]. Building on this, recent research has identified a “missing middle” in surface engineering—the meso-scale domain (10–500 µm)—which resides between conventional microroughness and macro-level design [23]. Within this range, features such as ridges, spikes, and grooves measuring between 40 µm and 200 µm are of particular interest as they align with the dimensions of native bone structures like osteons (100–300 µm), Haversian canals (20–100 µm), and Howship lacunae (20–80 µm). Unlike traditional microrough surfaces (1–5 µm) that typically enhance differentiation at the expense of cell proliferation, meso-scale architectures have been shown to promote both robust osteoblast attachment and rapid proliferation. Specifically, studies on zirconia and titanium featuring meso-scale spikes (e.g., 40–80 µm in width/height) [24,25] or laser-etched grids (40–140 µm) [26] demonstrate that these topographies provide critical spatial cues that guide multicellular organization and matrix deposition while simultaneously doubling the strength of bone integration through improved mechanical interlocking. In quantitative terms, a systematic review of 28 studies found that laser-modified titanium surfaces with meso-scale features (10–50 µm) either maintained or improved osteoblast adhesion and growth compared to standard surfaces in the majority of cases [23]. Studies on porous titanium architectures with controlled porosity (17–58%) further demonstrated that pore sizes ≥ 200 µm in the meso-scale range were required for osteoblast infiltration, whereas pores < 150 µm were consistently bridged over by cells without ingrowth [23]. Conversely, conventional microrough implant surfaces (Sa 1–5 µm) reduce osteoblast proliferation to approximately one-third to one-fifth of the rates observed on smooth machined titanium surfaces [27], underscoring the biological trade-off that meso-scale design strategies aim to overcome.

Compared to conventional subtractive manufacturing techniques such as computer-aided design/computer-aided manufacturing (CAD/CAM) milling, additive manufacturing enables the direct and reproducible fabrication of complex surface geometries with high spatial resolution. While milling processes are well established for zirconia fabrication, they are inherently limited in their ability to generate defined surface features beyond macro-scale geometries. In particular, the introduction of controlled meso-scale structures (10–500 µm) is technically challenging using subtractive approaches, as tool diameter, tool accessibility, and machining-induced stresses constrain geometric freedom.

Furthermore, the generation of micro- and nano-scale surface features via milling is virtually infeasible and typically requires additional post-processing steps such as sandblasting, acid etching, or laser treatment to achieve biologically favorable surface characteristics and promote osseointegration [28]. These secondary processes introduce stochastic surface characteristics and may compromise reproducibility, thereby limiting systematic investigation of structure–function relationships.

In contrast, additive manufacturing, particularly Digital Light Processing (DLP), facilitates a layer-by-layer fabrication process that allows precise and independent control over feature size, morphology, and spatial distribution across multiple length scales. This enables the intentional design and integration of meso-scale topographical elements directly into the component during fabrication, without the need for subsequent surface modification [12]. Thus, unlike subtractive approaches, where meso-scale structuring is limited and may compromise material integrity, additive manufacturing allows for controlled and reproducible integration of such features while preserving structural continuity.

While the biological relevance of intentionally designed meso-scale structures on zirconia remains incompletely understood, it is reasonable to hypothesize that such features may modulate early osteoblast responses at the cell–material interface in addition to their mechanical contribution.

Previous in vitro studies of this research group have demonstrated that osteoblast adhesion and proliferation on additively manufactured zirconia are comparable to those observed on conventionally milled zirconia. Moreover, unmodified additively manufactured surfaces have been reported to show slightly increased expression of early osteogenic markers such as alkaline phosphatase (ALPL) and runt-related transcription factor 2 (RUNX2), suggesting a preserved or potentially enhanced osteogenic differentiation potential [29,30]. However, these investigations relied on experimental or non-clinically approved zirconia materials, limiting direct clinical translation.

In contrast to our previous studies, the present investigation employs zirconia materials approved for clinical use, enabling a biologically and translationally relevant assessment of additively manufactured zirconia surfaces. By leveraging the design freedom of additive manufacturing, meso-scale modifications were intentionally incorporated into the zirconia surfaces to explore their potential mechanical and biological implications.

It is therefore hypothesized that the intentionally introduced meso-scale structures on additively manufactured zirconia surfaces may not only improve primary stability from a biomechanical standpoint, but also exert biologically relevant effects on osteoblast adhesion, proliferation, and differentiation toward an osteoblastic phenotype.

Thus, the aim of the present study was to investigate the interaction of osteoblasts with additively manufactured, clinically approved zirconia, with particular emphasis on cell adhesion, proliferation, and differentiation toward an osteoblastic phenotype. By directly comparing the biological performance of additively fabricated zirconia surfaces and considering their inherent surface characteristics, this study aims to deepen our understanding of the osseointegration potential of 3D-printed zirconia. While in vitro experimental models are a fundamental and widely accepted approach for investigating early cell–material interactions under controlled conditions [31,32], their limitations must be acknowledged. Specifically, although osteoblast adhesion, proliferation, and early differentiation are highly sensitive to surface cues and can be reliably assessed in vitro—serving as predictive indicators of biomaterial performance, providing essential mechanistic insights, and acting as a necessary preclinical screening step before animal and clinical studies—such findings cannot replicate the complexity of osseointegration in vivo.

## 2. Materials and Methods

### 2.1. Study Design

**Figure 1:**
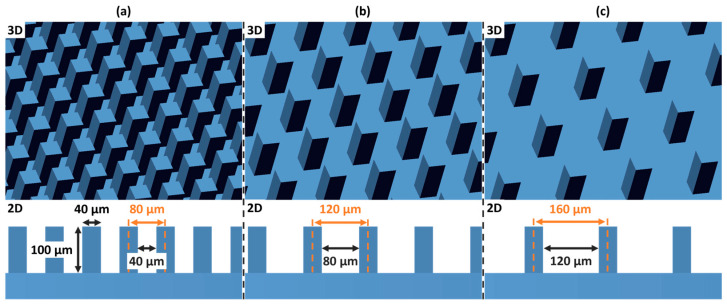
Three-dimensional (3D, **top**) and two-dimensional (3D, **bottom**) view of the surface modifications on planar samples of the as-print state: (**a**) Mod-80, (**b**) Mod-120 and (**c**) Mod-160 with numbers referring to the grid distance. It is important to note that the specified dimensions refer to the as-printed state, prior to debinding and sintering, and therefore do not account for subsequent sintering shrinkage. The vertical height of the pillars of 100 µm refers to 2 printing layers.

This in vitro study investigated the influence of material composition and meso-scale topographical modifications on the cellular response—specifically adhesion, proliferation, and differentiation—of osteoblastic cells. Research was conducted on additively manufactured zirconia, specifically 3 mol% yttria-stabilized tetragonal zirconia polycrystal (3Y-TZP) and 5 mol% yttria partially stabilized zirconia (5Y-PSZ). Beyond the inherent material properties, a primary focus of this investigation was the impact of defined meso-scale surface manipulations on cellular behavior. For each material, specimens were divided into four groups: a planar control group without structural modification, and three experimental groups featuring regularly distributed meso-scale columnar structures. The three experimental groups were designed with regularly distributed columnar surface features of 100 µm height and quadratic cross-section with an edge length of 40 µm. These experimental groups differed only in the center-to-center separation distance between the columns, which was set at 80 µm, 120 µm, and 160 µm, respectively. The three different surface meso-scale modifications are illustrated in Figure 1. From here on, these are referred to corresponding to the planned respective center-to-center distance between their columns: Mod-80, Mod-120, and Mod-160.

**Figure 2:**
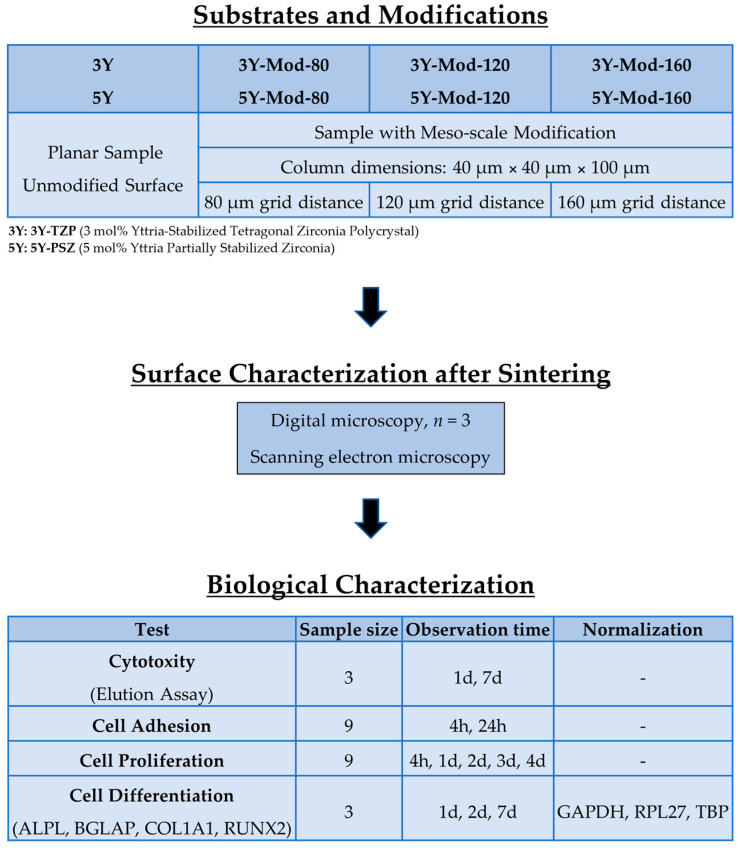
Experimental workflow for the evaluation of meso-scale modified zirconia substrates. All indicated sample sizes (*n*) are specified per group.

The study workflow is depicted in Figure 2.

### 2.2. Specimen Fabrication

Surface meso-scale modifications were designed using CAD software (Geomagic Design X V2023; Hexagon AB, Stockholm, Sweden). The column width, height and separation distances were selected in accordance with the manufacturing capabilities of the 3D-printing system (Zipro-D Dental and Zipro Dental; AON Co., Ltd., Seoul, Republic of Korea) for horizontally nested samples. The printer’s horizontal resolution, characterized by a minimum feature size of 40 µm, determined both the column width and the spacing between adjacent columns. The vertical resolution, defined by a layer thickness of 50 µm, governed the column height, such that a height of 100 µm required the deposition of two successive layers to form the meso-scale modifications. Cylinders with a diameter of 5 or 12 mm (depending on the respective test requirements) were scaled according to the expected shrinkage and modified as shown above. Cylinder thickness was set to 1 mm in the as-print state.

Data was then transferred to the printing system’s’ preprocessing software (ZiproS V3.1.106; AON), and slicing was performed with a layer thickness of 50 µm. Additive manufacturing of the zirconia specimens was performed using digital light processing (DLP) technology. The 3Y-TZP samples were fabricated from the ceramic suspension InniCera BCM W1000 (AON) using the Zipro-D Dental printer (AON), whereas the 5Y-PSZ samples were produced from the corresponding InniCera BCM W1000-T (AON) suspension using the Zipro Dental printer (AON). The printing processes took place with parameters predefined by the manufacturer. After printing, all specimens were cleaned from excess slurry with an airbrush at 0.1 MPa and isopropanol as a solvent. Debinding and pre-sintering were conducted for 30 h at up to 1100 °C (ZIRFUR, AON), and final sintering was carried out at up to 1500 °C for 6 h (SINTRA PRO/120zrf, Shenpaz Dental Ltd., Migdal HaEmek, Israel).

#### Specimen Characterization

After final sintering, specimen surface structures were characterized using a digital microscope (Smartzoom5, Zeiss, Jena, Germany) equipped with an extended depth of field (EDF) function. For each area, a series of images was acquired at successive focal points along the vertical axis with a step size of 0.5 µm. These images were computationally combined into an all-in-focus EDF image. Based on the known vertical position of each focal plane and the corresponding in-focus pixel information used during EDF reconstruction, a height map was generated. Profile measurements were then taken from these reconstructed 3D surfaces to quantify the geometry of the surface features. Measurements were conducted on one representative specimen per group at three different locations on the sample surface at 1000× magnification. At each location, two measurements of the center-to-center separation distance and the column height were conducted.

Scanning electron microscope (SEM) images (JSM-6510, JEOL, Freising, Germany) were obtained on an exemplary specimen of group 5Y-Mod-160 at 500× magnification and 30° tilt after gold sputtering (Cressington 108auto, Cressington Scientific Instruments Ltd., Watford, UK) to further analyze the fabricated surface modification.

### 2.3. Analysis of Cell–Material Interactions

#### 2.3.1. Cell Culture

The experimental procedures intend the analysis of cell–material interactions utilized the human fetal osteoblast cell line hFOB 1.19 (CRL-11372; American Type Culture Collection, ATCC^®^, Manassas, VA, USA). The basal growth environment consisted of a 1:1 (*v*/*v*) ratio of Ham’s F-12 Medium and Dulbecco’s Modified Eagle Medium (DMEM) enriched with 2.5 mM L-glutamine. The medium was supplemented with 10% fetal bovine serum (FBS), 100 IU/mL penicillin, 100 μg/mL streptomycin, 2.5 mg/mL amphotericin B, and 300 μg/mL G418 (Bio&Sell, Feucht, Germany). Cultivation was performed under controlled conditions within a humidified atmosphere containing 5% CO_2_ at the permissive temperature of 34 °C.

The L929 murine fibroblast cells (CCL-1, ATCC, Manassas, VA, USA), which served as a standardized system for evaluating the potential cytotoxic effects of the zirconia specimen, were cultivated in Dulbecco’s Modified Eagle Medium (DMEM) enriched with 2.5 mM L-glutamine. The medium was supplemented with 10% fetal bovine serum (FBS), 100 IU/mL penicillin, 100 μg/mL streptomycin, and 2.5 mg/mL amphotericin B (Bio&Sell, Feucht, Germany) and cultivated within a humidified atmosphere containing 5% CO_2_ at 37 °C. The L929 murine fibroblast cell line is widely recommended for cytotoxicity testing in accordance with ISO 10993-5 [33] due to its high sensitivity to leachable toxic substances and reproducible growth characteristics. Its use enables standardized comparison across biomaterials and ensures compliance with regulatory requirements for biocompatibility assessment.

#### 2.3.2. Control Surfaces/Tissue Culture Plastic

Standardized polystyrene tissue culture surfaces served as the control group for the comparative assessment of cellular attachment, confluent coverage, and osteogenic differentiation. All requisite polymeric culture vessels—including expansion flasks, Petri dishes, and multi-well assay plates—were sourced from Greiner Bio-One GmbH (Frickenhausen, Germany). These materials were utilized in strict accordance with the manufacturer’s technical specifications.

#### 2.3.3. Indirect Cytotoxicity Assessment via Elution Analysis

To evaluate the potential release of cytotoxic substances from the zirconia specimens, an elution assay was conducted in accordance with the specifications outlined in ISO 10993-5.

##### Preparation of Extracts

Specimens possessing a standardized surface area of 3 cm^2^ were submerged in 1 mL of unsupplemented Dulbecco’s Modified Eagle Medium (DMEM) (Bio&Sell, Feucht, Germany). Extraction was performed under static conditions for durations of 1 day and 7 days to capture both immediate and sustained leachability profiles. Following the respective elution periods, the extracts were harvested for biological testing.

##### Biological Testing

Murine L929 fibroblasts (CCL-1, ATCC, Manassas, VA, USA) were utilized as the indicator cell line for the cytotoxicity screening. The cells were exposed to the resulting eluates, and their viability was quantified using the colorimetric CellTiter 96^®^ assay (Promega, Mannheim, Germany). For each experimental group and time point, three independent specimens were evaluated (*n* = 3). Cells grown in standard DMEM served as controls.

#### 2.3.4. Cell Adhesion and Proliferation

The adhesive and proliferative behaviors of hFOB 1.19 cells on zirconia substrates were quantified using the CellTiter 96^®^ metabolic activity assay (Promega, Mannheim, Germany). Zirconia specimens were positioned in 96-well plates and inoculated with a density of 10,000 cells per well in 200 µL of culture medium. Incubation was conducted at 34 °C under a 5% CO_2_ atmosphere.

##### Cytoadhesion

To evaluate initial attachment, cells were cultured for 4 h and 24 h. Given that the specimen diameter was smaller than the well diameter, a transfer protocol was implemented to ensure that only cells adhered to the zirconia surfaces were measured. Specimens were precisely extracted using Dumont forceps (#7, tip size 0.07 × 0.04 mm^2^, F.S.T., Heidelberg, Germany), ensuring no contact with the superior test surface, and relocated to a secondary plate containing 100 µL of medium. Following the manufacturer’s protocol, optical density (OD) was recorded at 490 nm using a GeniosPro multi-well reader (Tecan, Crailsheim, Germany). Relative adhesion was calculated as the ratio of specimen OD to positive control OD (cells on tissue culture plastic, TCP), adjusted for surface area differences, with TCP defined as 100%. Nine surfaces or controls (biological replicates) were utilized for each condition.

##### Proliferation Kinetics

Cellular expansion was monitored at 4 h and then daily for 4 days post-seeding. For the longitudinal analysis of growth, the 4 h measurement was designated as the 100% baseline. Nine individual surface specimens or controls (biological replicates) were utilized for each condition and time point. The use of growth curve slopes allows for the comparison of proliferation kinetics independent of initial cell seeding density and adhesion differences. This approach provides a more accurate representation of cell division rates over time compared to single time point measurements, particularly in systems where early adhesion varies between experimental groups.

#### 2.3.5. RT-PCR Analysis

To evaluate the osteogenic transcriptional profile, hFOB 1.19 cells were cultivated on zirconia specimens or within standard polystyrene wells (*n* = 12 per cohort). To achieve an adequate nucleic acid yield for downstream applications, total RNA was isolated from the consolidated biomass of four pooled replicates per condition utilizing the RNeasy Kit (Qiagen, Hilden, Germany), so that three independent samples were analyzed. The structural integrity and purity of the harvested RNA were validated through automated capillary electrophoresis using the Experion System (Bio-Rad, Munich, Germany).

##### cDNA Synthesis and Azure Cielo Amplification

Purified RNA was reverse-transcribed into complementary DNA (cDNA) employing recombinant Moloney Murine Leukemia Virus Reverse Transcriptase (M-MuLV RT) and poly-dT priming (Thermo Fisher Scientific, Dreieich, Germany). The subsequent quantitative PCR (qPCR) was executed on the Azure Cielo real-time PCR platform (Azure Biosystems, Biozym, Oldendorf, Germany) utilizing target-specific primer-probe chemistry (Bio-Rad, Munich, Germany). All oligonucleotides and fluorogenic probes were synthesized by Merck (Darmstadt, Germany); detailed sequences for the primers and probes are cataloged in Table 1.

##### Normalization and Data Quantification

Amplification efficiency for each primer-probe set was monitored through serial dilution-derived analyses. In instances where efficiencies deviated, mathematical compensation was applied to the quantification model. Three independent internal reference genes—Glyceraldehyde 3-phosphate dehydrogenase (GAPDH), TATA-binding protein (TBP), and Ribosomal protein L27 (RPL27)—were analyzed. The geometric mean of these housekeeping genes was calculated to establish a stable normalization factor [34]. Relative fold changes in gene expression were subsequently determined via the comparative threshold cycle delta-delta CT (ΔΔCT)) method [35]. Each experimental condition was processed in technical triplicates. Three independent biological samples per condition (each representing pooled RNA from four specimens) were analyzed (*n* = 3). Given the resulting sample size, the gene expression data were conceived as exploratory and hypothesis-generating, and the observed material- and geometry-dependent effects are interpreted with appropriate caution.

## 3. Results

### 3.1. Meso-Scale Topography Characterization

**Figure 3:**
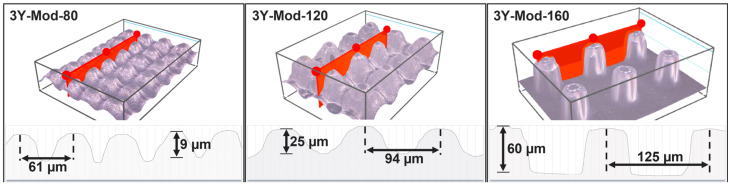
Representative profile measurements of all three surface modalities. For clarity, only images of 3Y-TZP are shown. Illustrated are column heights and center-to-center separation distances.

Figure 3 shows an exemplary image for each surface modality and material, with the mean center-to-center separation distances and column heights being marked.

Center-to-center separation distances (mean value ± standard deviation) for the 3Y-Mod-80/120/160 specimens amounted to 61 µm ± 2 µm, 94 µm ± 2 µm and 125 µm ± 1 µm, respectively. Similarly, measurements of 5Y-Mod-80/120/160 revealed separation distances of 62 µm ± 2 µm, 97 µm ± 3 µm and 129 µm ± 1 µm. This is in accordance with the expected horizontal shrinkage that occurs during sintering by factors of 1.288 for 3Y-TZP and about 1.257 for the 5Y-PSZ specimen. Shrinkage factor determination is thoroughly described by Rues et al. [36].

Column height found in the printed samples strongly varies between the groups and is predominantly affected by the modality of the specimen and the zirconia type it was fabricated with. The lowest column height was seen for 5Y-Mod-80 with 5 µm ± 1 µm, followed by 3Y-Mod-80 with 9 µm ± 1 µm, 5Y-Mod-120 with 20 µm ± 2 µm, 3Y-Mod-120 with 25 µm ± 2 µm, 5Y-Mod-160 with 57 µm ± 12 µm and 3Y-Mod-160 60 µm ± 2 µm. Vertical shrinkage factors amounted to 1.358 for 3Y-TZP and 1.307 for 5Y-PSZ.

Despite the systematic offset between the nominal CAD geometry and the sintered structures, center-to-center distances and column heights exhibited relatively low intra-group variability, as reflected by standard deviations of 1–3 µm for lateral spacing and 1–12 µm for column height. This indicates that, once the material- and process-specific shrinkage and overgrowth behavior are characterized, the resulting meso-scale topographies can be reproduced with reasonable fidelity within each modification group, albeit at dimensions that differ from the original CAD input.

### 3.2. Biological Characterization of Zirconia Surfaces

#### 3.2.1. Cytotoxicity of 3D Printed Zirconia

**Figure 4:**
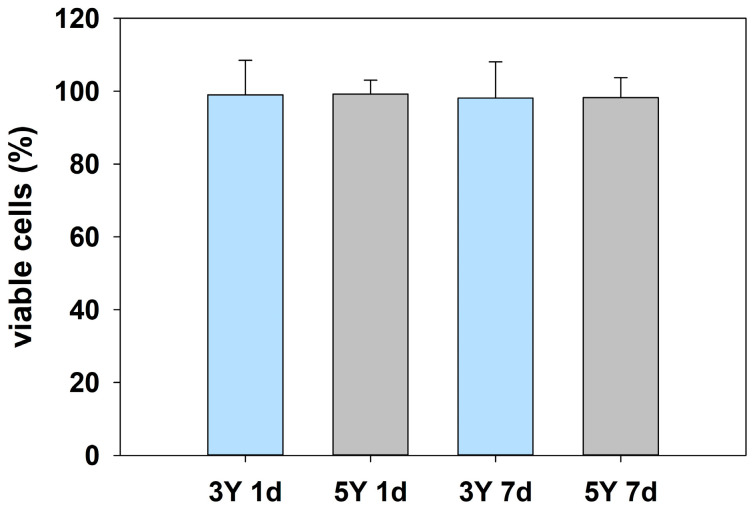
Cytotoxicity of DLP-printed zirconia specimens assessed by a standard elution test. Cell viability after 1 day (1 d) and 7 days (7 d) of elution is shown relative to untreated control cells, which were set to 100% survival. Both 3Y and 5Y zirconia exhibited survival rates comparable to the control, with no statistically significant differences between materials, elution times, or the control group, indicating the absence of cytotoxic effects.

Despite the clinical approval of the investigated DLP-printed zirconia materials, verification of their cytotoxicity under realistic laboratory conditions remains essential to confirm that the applied manufacturing workflow, including printing and post-processing steps, is adequate and results in a material performance consistent with the documented approval. Therefore, cytotoxicity was assessed using a standard elution test with extraction periods of 1 day (1 d) and 7 days (7 d). Cell viability was determined relative to untreated control cells, which were defined as 100% survival. All tested specimens showed survival rates comparable to the control group, ranging from 99.19% for 5Y zirconia after 1 d elution to 98.1% for 3Y zirconia after 7 d elution (Figure 4). No statistically significant differences were observed between either zirconia material and the control for any elution time, nor between the two materials themselves, indicating the absence of cytotoxic effects under the tested conditions.

Specimens featuring meso-scale surface modifications were deliberately excluded from cytotoxicity testing. Although such modifications may theoretically increase the overall surface area, no relevant differences in cytotoxic response were anticipated. Furthermore, cylindrical specimen geometry would have limited meso-scale structural alterations to the external surface only, resulting in a comparatively small proportion of modified area relative to the total specimen volume. Inclusion of these specimens would also have impeded accurate surface area determination, thereby compromising the comparability and interpretability of the elution-based cytotoxicity results.

#### 3.2.2. Cell Adhesion

**Figure 5:**
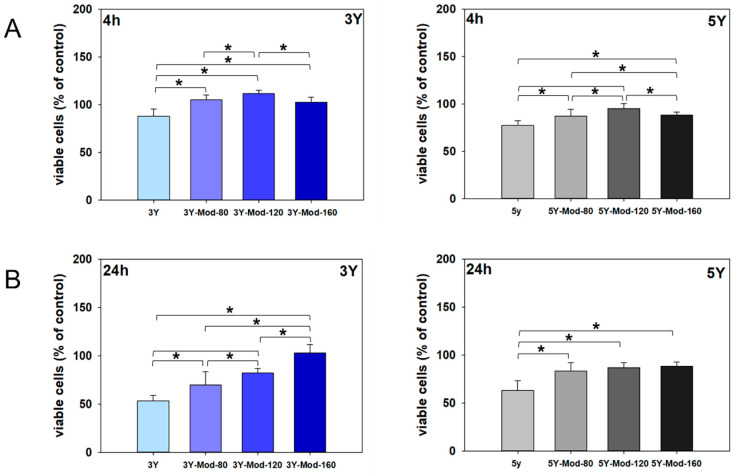
Adhesion of hFOB 1.19 cells on DLP-printed 3Y and 5Y zirconia surfaces with and without meso-scale modifications. Cell adhesion after 4 h (**A**) and 24 h (**B**) is shown relative to tissue culture plastic (TCP), which was set to 100%. Meso-scale modified surfaces (Mod-80, Mod-120, Mod-160) generally exhibited significantly higher cell adhesion compared with unmodified zirconia for both 3Y and 5Y materials, with differences becoming more pronounced after 24 h. A reduction in cell adhesion from 4 h to 24 h was observed mainly on unmodified and Mod-80 and Mod-120 3Y surfaces, whereas Mod-160 3Y and 5Y surfaces sustained cell adhesion over time. Significant differences were also detected between specific meso-scale feature sizes, indicating an influence of meso-scale structural design on the maintenance of cell adhesion. Significant differences between groups are indicated by square brackets; for clarity, asterisks denoting statistical significance were omitted. Kruskal–Wallis One-Way Analysis of Variance on Ranks, Bonferroni post hoc test, *n* = 9.

Adhesion of hFOB 1.19 cells to the zirconia surfaces was quantified after 4 h and 24 h and expressed relative to adhesion on tissue culture plastic, which was arbitrarily set to 100%. A general decrease in cell adhesion from 4 h to 24 h was observed, particularly on 3Y zirconia surfaces. Specifically, cell adhesion decreased by 34.4% on unmodified 3Y, by 35.5% on 3Y-Mod-80, and by 29.5% on the 3Y-Mod-120 specimen. Interestingly, no reduction in cell adhesion was detected on the 3Y-Mod-160 specimen over time. A comparable trend of reduced adhesion between 4 h and 24 h was also observed for 5Y zirconia, although the extent of reduction was markedly less pronounced. Notably, 5Y-Mod-120 zirconia similarly sustained cell adhesion from 4 h to 24 h. Results are illustrated in Figure 5.

Overall, cell adhesion at both 4 h and 24 h was significantly higher on meso-scale structurally modified surfaces of both 3Y and 5Y zirconia compared to the respective unmodified surfaces, resulting in significant differences between unmodified 3Y and 5Y and all meso-scale structural modifications (Mod-80, Mod-120 and Mod-160). These differences were even more pronounced after 24 h for both zirconia types. In addition, significant differences were observed between Mod-120 and Mod-40 as well as Mod-160 specimen for 3Y and 5Y zirconia after 4 h. After 24 h, 3Y-Mod-160 supported significantly higher cell adhesion than 3Y-Mod-80 and 3Y-Mod-120. Taken together, these findings suggest that meso-scale surface modifications may positively influence, in particular, the maintenance of hFOB 1.19 cell adhesion over time when compared with unmodified DLP-printed zirconia specimens of both 3Y and 5Y composition.

#### 3.2.3. Cell Proliferation

**Figure 6:**
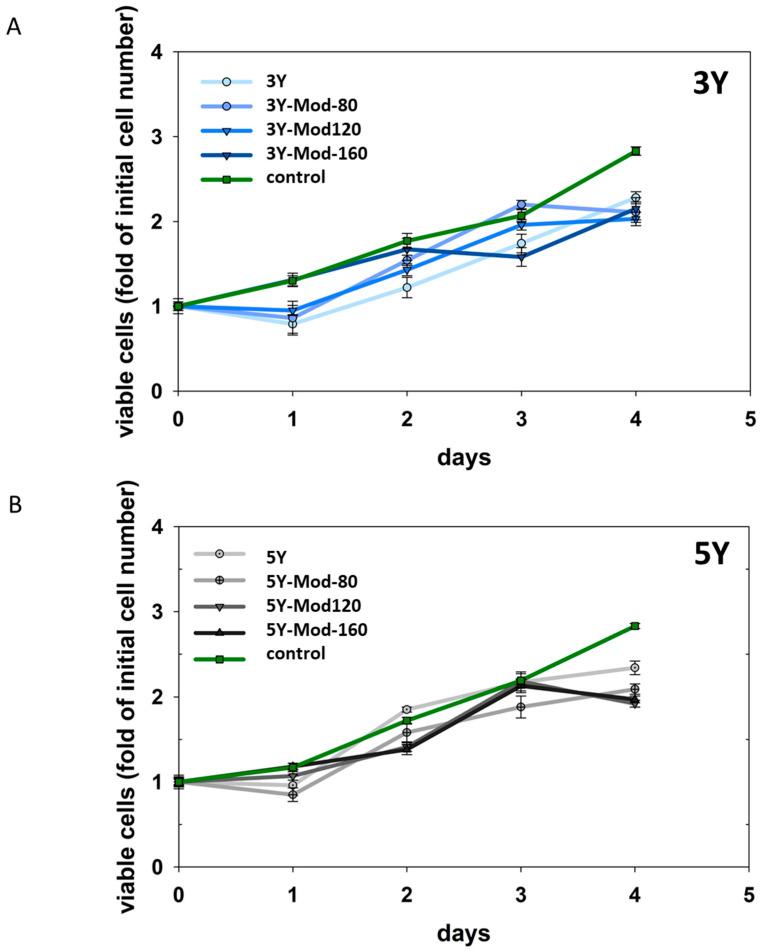
Proliferation of hFOB 1.19 cells on DLP-printed 3Y (**A**) and 5Y (**B**) zirconia surfaces with and without meso-scale structural modifications. Cell numbers were determined after 4 h (day 0) and daily for a total of 4 days. Growth curves are shown for unmodified and meso-scale structurally modified surfaces (Mod-80, Mod-120, Mod-160) and compared with cells cultured on standard tissue culture plastic (control). No significant differences in proliferation rates, assessed by comparison of growth curve slopes, were observed between surface modifications, zirconia materials, or the control, indicating comparable cell growth on all tested surfaces.

Proliferation of hFOB 1.19 cells on the investigated zirconia surfaces, including meso-scale structurally modified specimens, was monitored over a period of four days. Cell numbers were determined after 4 h (defined as day 0) and subsequently on a daily basis. To assess potential differences in proliferation behavior, growth rates were compared by analyzing the slopes of the respective growth curves. No statistically significant differences in growth rates were detected between the different surface modifications, nor between 3Y and 5Y zirconia. Notably, growth rates on all zirconia surfaces were also comparable to those observed for the control condition, i.e., cells cultured on standard tissue culture plastic. Proliferation results are illustrated in Figure 6.

Although higher cell numbers were observed on meso-scale structurally modified surfaces—particularly on Mod-160 zirconia—after 24 h (see cell adhesion results), these differences were most likely attributable to enhanced initial cell adhesion rather than to increased proliferation between 4 h and 24 h. This interpretation is supported by the comparable proliferation rates observed across all surface modifications and both zirconia materials throughout the observation period. Overall, meso-scale surface modifications did not appear to influence hFOB 1.19 cell proliferation. Importantly, the absence of significant differences in growth rates compared with standard tissue culture plastic suggests that cell proliferation was sufficiently sustained and not markedly impaired on either 3Y or 5Y DLP-printed zirconia, irrespective of surface modification.

#### 3.2.4. Osteoblastic Gene Expression Analysis

To evaluate the potential influence of zirconia surface topography on osteogenic differentiation of hFOB 1.19 cells, gene expression analyses were performed under differentiation-permissive conditions. Cells were cultured at 39 °C, corresponding to inactivation of the temperature-sensitive large T antigen, and harvested after 1, 2, and 7 days. The expression of key osteogenic marker genes representing different stages of osteoblast differentiation was quantified by real-time PCR. Osteogenic differentiation is a multistep process that involves commitment of osteoprogenitor cells, extracellular matrix production, matrix maturation, and finally mineralization. Runt-related transcription factor 2 (RUNX2) is a master transcription factor required for early osteoblast commitment, collagen type I alpha 1 chain (COL1A1) reflects matrix synthesis during early to intermediate differentiation, alkaline phosphatase, tissue-nonspecific isozyme (ALPL) is associated with matrix maturation and early mineralization, and osteocalcin (bone gamma-carboxyglutamate protein; BGLAP) is a late marker indicative of terminal osteoblast differentiation. Together, these markers allow assessment of the differentiation stage of hFOB 1.19 cells cultured on zirconia surfaces.

On 3Y zirconia, a significant early reduction in RUNX2 expression was observed on meso-scale structurally modified surfaces compared with the unmodified control, starting as early as day 1. This decrease became more pronounced with increasing feature size, showing a gradual reduction from Mod-80 to Mod-160 µm modifications. At day 2, RUNX2 expression levels were largely comparable among the surfaces, whereas at day 7, a marked and significant downregulation was detected specifically on the Mod-160 surface. ALPL expression similarly exhibited a significant decrease at day 1 on all meso-scale structurally modified surfaces compared with unmodified 3Y zirconia. No significant differences were detected at day 2; however, at day 7, ALPL expression was significantly reduced on the Mod-80 and Mod-120 surfaces and moderately decreased on the Mod-160 surface relative to the unmodified control. In contrast, COL1A1 expression was not significantly affected by meso-scale structural modification at any time point, although a slight trend toward increased expression was evident at day 7 for all modified surfaces compared with unmodified zirconia.

Most notably, expression of the late osteogenic marker BGLAP was induced early and strongly on meso-scale structurally modified surfaces. On the Mod-160 surface, BGLAP expression was significantly increased as early as day 1 compared with the 40 µm modification. At days 2 and 7, BGLAP expression on the Mod-160 surface remained significantly higher than on the Mod-80 and Mod-120 modifications, as well as the unmodified 3Y surface. These findings suggest that meso-scale modification, particularly with Mod-160 features, may promote a shift toward terminal osteogenic differentiation of hFOB 1.19 cells on 3Y zirconia.

On 5Y zirconia surfaces, RUNX2 expression exhibited a pattern broadly comparable to that observed on 3Y zirconia, although the magnitude of the effects was markedly attenuated. Overall, RUNX2 expression tended to be reduced on meso-scale structurally modified surfaces relative to unmodified 5Y zirconia; however, this reduction was less pronounced than on 3Y surfaces. Interestingly, a transient and significant increase in RUNX2 expression was detected at day 2 when comparing the Mod-80 and Mod-120 modifications. A similar pattern was observed at day 7, where RUNX2 expression on the Mod-80-modified surface was significantly higher than on the Mod-120 and Mod-160 modifications. Despite these relative increases among modified surfaces, RUNX2 expression on the Mod-160 5Y surface was significantly reduced compared with the unmodified control at day 7, mirroring the trend observed for 3Y zirconia, albeit to a substantially lesser extent.

In contrast to the pronounced changes observed on 3Y zirconia, ALPL expression on 5Y surfaces was not significantly affected by meso-scale structural modifications at any of the investigated time points. Nevertheless, a slight tendency toward increased ALPL expression was apparent at day 7 on meso-scale structurally modified surfaces, which is opposite to the reduction in ALPL expression observed on modified 3Y zirconia. Similarly, COL1A1 expression did not differ significantly between unmodified and meso-scale structurally modified 5Y surfaces at any time point. As for 3Y zirconia, a modest trend toward higher COL1A1 expression on modified surfaces was evident, particularly at later stages of culture.

**Figure 7:**
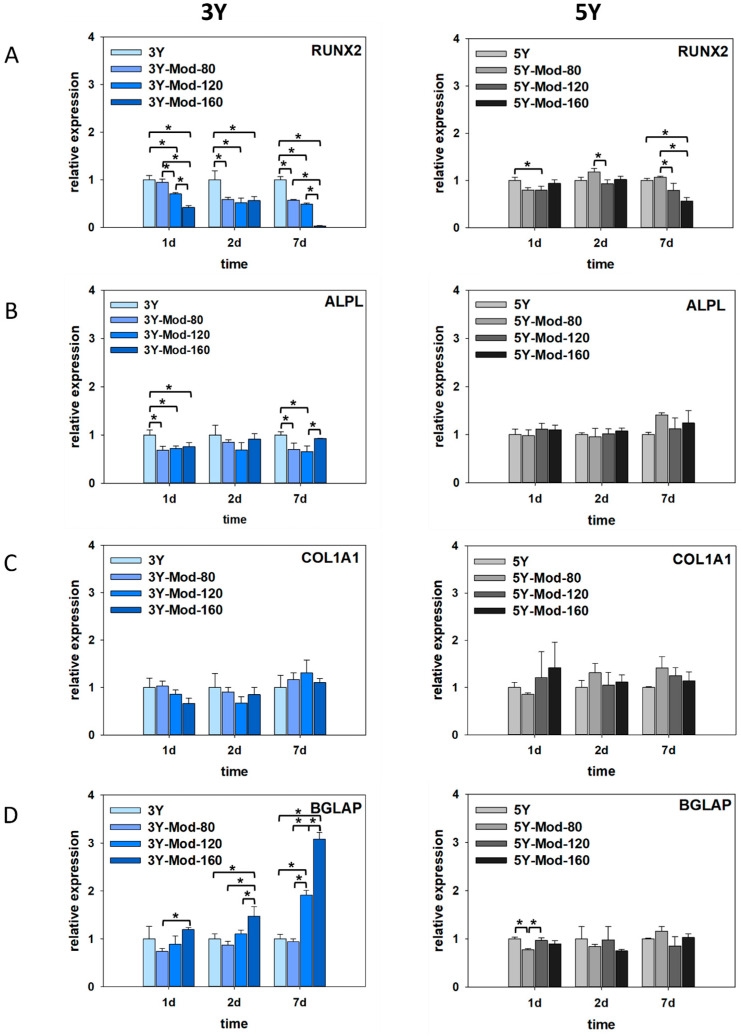
Osteogenic differentiation-associated gene expression of hFOB 1.19 cells cultured on DLP-printed 3Y and 5Y zirconia surfaces with and without meso-scale structural modifications. Cells were analyzed after 1, 2, and 7 days. Relative mRNA expression levels of early (RUNX2, (**A**)), intermediate (ALPL, (**B**); COL1A1, (**C**)), and late (BGLAP, (**D**)) osteogenic markers were quantified by qPCR. Meso-scale structural modifications (Mod-80, Mod-120 and Mod-160) induced material- and feature size-dependent differences in gene expression, with pronounced early and sustained induction of BGLAP on modified 3Y zirconia, particularly on Mod-160, whereas 5Y zirconia showed more moderate or absent differentiation-related responses. Significant differences between groups are indicated by square brackets; asterisks were omitted for clarity. Kruskal–Wallis One-Way Analysis of Variance on Ranks, Bonferroni post hoc test, *n* = 3.

In marked contrast to the findings on 3Y zirconia, BGLAP expression on 5Y surfaces was not significantly induced by meso-scale modifications. Instead, a significant decrease in BGLAP expression was observed at day 1 on the Mod-80 surface compared with both the Mod-120 and unmodified 5Y surfaces. At later time points, no significant upregulation of this late osteogenic marker was detected on any of the modified 5Y surfaces. Collectively, these results indicate that meso-scale surface modifications exerted a distinct and material-dependent influence on osteogenic gene expression. While meso-scale structurally modification of 3Y zirconia, particularly with Mod-160 features, appeared to promote expression of genes associated with terminal osteoblast differentiation, the corresponding modifications on 5Y zirconia did not elicit a similar molecular response. This divergence may suggest a less inductive stimulus for osteogenic differentiation on modified 5Y surfaces at the mRNA level, which could be advantageous in clinical scenarios where sustained proliferative capacity of osteoblasts is desired. Conversely, the stronger induction of BGLAP expression on modified 3Y zirconia may be beneficial in situations where accelerated osteogenic differentiation is advantageous. Results are illustrated in Figure 7.

## 4. Discussion

### 4.1. Meso-Scale Topography

As seen in Figure 3, column heights and the areas between the columns strongly differ from the designed geometry that is illustrated in Figure 1a–c, even after accounting for horizontal and vertical shrinkage during sintering. A primary cause of this deviation is the overlap of adjacent columns resulting from light scattering during photopolymerization in the printing process. Scattered light leads to unintended curing beyond the nominal exposure area, producing columns with a larger effective width than the designated 40 µm. Consequently, neighboring columns partially merge at their bases, which reduces the measurable separation distance between columns—if a distinct boundary can be defined at all—and limits the achievable column height.

**Figure 8:**
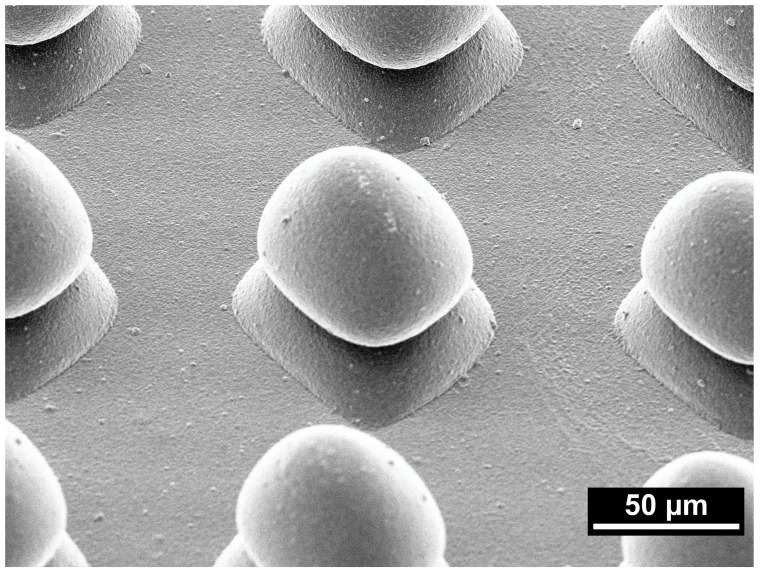
Representative scanning electron microscope image of a 5Y-Mod-160 sample under 30° tilt and 500× magnification.

Figure 8 shows a representative SEM image of a 5Y-Mod-160 sample under 30° tilt and illustrates the overgrowth of the illuminated area. Columns of this modification are separated sufficiently far; therefore, no column overlap is observed.

Smaller separation distances between the columns lead to an increased column overlap, which is consistent with the smallest observed column heights for the Mod-80 specimen. Differences in refractive indices between the ceramic particles and the photocurable resin are the primary cause for light scattering in ceramic suspensions [37,38,39,40], and it was shown that exposure time and exposed area influence its magnitude [39,41]. Internal investigations on macro-scale calibration objects [36] revealed that the overgrowth effect is more pronounced in the 5Y-PSZ suspension, which causes the smaller column height for 5Y-Mod-80/120/160 compared to the 3Y-modifications. During fabrication of typical prosthodontic restorations, this effect can be factored in by subtracting an offset value from the exposed area. Since only one voxel is illuminated during the fabrication of the different meso-scale modifications in this study, a subtraction of exposed area would result in no illumination and therefore could not be applied here. Taken together, the mismatch between designed and realized geometries should be regarded less as a random manufacturing error and more as a systematic, process-inherent distortion that can be quantified and, in principle, compensated for in future design cycles. Nevertheless, the limited absolute design fidelity at the meso-scale represents a current constraint of ceramic DLP printing and underscores the need for refined process models and correction algorithms before these structures can be translated into tightly specified clinical implant surfaces.

Figure 3 and Figure 8 revealed that the Mod-160 specimens of both materials show no apparent overlap between adjacent columns, yet their column heights still do not correspond to the nominal height after sintering (74 µm for 3Y-TZP and 77 µm for 5Y-PSZ samples). Although the precise reasoning cannot be clarified, a possible explanation could be insufficient removal of excess suspension between the columns, as the areas between the columns are rather small.

### 4.2. Adhesion

Cell adhesion to ceramic biomaterials is mediated by the adsorption of serum proteins such as fibronectin, vitronectin, and albumin, which form a conditioning layer on the material surface. Osteoblasts interact with these proteins via integrin receptors, leading to the formation of focal adhesions [42]. This process activates intracellular signaling pathways, including focal adhesion kinase (FAK) and downstream MAPK/ERK cascades, which regulate cytoskeletal organization, cell survival, and differentiation. Surface properties such as roughness, wettability, and chemistry strongly influence protein adsorption kinetics and conformation, thereby modulating subsequent cellular responses. Previous studies demonstrated that meso-architectural features enhance osteoblast adhesion and promote homogeneous cell distribution by recreating a three-dimensional extracellular matrix (ECM)-like microenvironment [24,43]. In particular, Meso-scale topographical features (10–500 µm) influence osteoblast adhesion and spreading by interacting with cells at the level of whole-cell morphology and multicellular organization. Osteoblasts typically spread to dimensions of approximately 20–50 µm, placing them directly within this topographical range and enabling spatial guidance through geometric constraints [23,44]. In contrast to micro-scale roughness, which primarily regulates subcellular adhesion processes, meso-scale features provide contact guidance cues that influence cell alignment and collective organization [45,46]. Furthermore, these structures mimic native bone architecture, such as osteons and trabecular elements, which exist within the same dimensional range and play a critical role in cell migration and tissue organization [47,48]. Experimental evidence suggests that such features enhance cell spreading, promote cytoskeletal organization, and facilitate extracellular matrix deposition, thereby improving early cell–material interactions [19]. Collectively, meso-scale topography acts as a biological and structural intermediary between micro-/nano-scale features and tissue-level organization, supporting more physiologically relevant osteogenic responses [23]. The present study aimed to gain insight into how such meso-topographical modifications influence early osteoblastic adhesion dynamics on DLP-printed zirconia surfaces. Most importantly, at both observation time points, at 4 h and 24 h, adhesion was significantly higher on all meso-scale modified surfaces compared with the respective unmodified controls, for both 3Y and 5Y zirconia. At the same time, our data reveal geometry-dependent nuances, suggesting that larger meso-features (Mod-160) may provide enhanced mechanical interlocking or improved spatial confinement, thereby stabilizing adhesion complexes [49]. This interpretation is consistent with previous work on bio-inspired meso–nano hybrid topographies on zirconia, where the introduction of meso-spike structures markedly increased surface roughness and effective surface area without compromising osteoblast adhesion after 24 h [24]. However, the differences between modifications (Mod-80, Mod-120, Mod-160), although statistically significant, were not dramatic in magnitude. This again indicates that the biological impact of meso-scale modifications is subtle and likely modulated by additional factors, including substructural surface morphology arising from DLP fabrication (see below).

### 4.3. Proliferation and Differentiation

In vitro evidence has established that surface topography across micro-, nano-, and meso-scales exerts distinct yet partially overlapping effects on osteoblastic behavior [27]. Only lately, meso-scale structures (10–500 μm) have increasingly been recognized as biologically active in their own right [23].

With respect to proliferation, our data demonstrate that meso-scale surface modifications on DLP-printed 3Y and 5Y zirconia did not significantly alter hFOB 1.19 cell growth compared with standard tissue culture plastic. Importantly, proliferation was not impaired on any of the tested surfaces, irrespective of the specific meso-scale geometry. Although we did not observe a statistically significant increase in proliferation induced by our meso-scale modifications, the absence of any growth inhibition is biologically meaningful. It indicates that meso-scale structuring—at least in the geometries and dimensions realized in our DLP-printed zirconia—does not reproduce the proliferation-suppressive effect classically associated with pronounced microroughness, and it suggests that additional anchorage can be provided without incurring a proliferation penalty. In this respect, our findings are broadly confirmatory of the concept: meso-scale roughness does not impede proliferation despite increased surface complexity [23].

At the same time, our results suggest that not all meso-scale modifications are equivalent. While differently sized modifications did not induce statistically distinct proliferation profiles in our model, differences in cell adhesion were observed. This aligns with the notion that meso-scale features primarily guide spatial organization and multicellular arrangement rather than directly accelerating cell cycle progression.

Regarding our differentiation analyses, the overall pattern of results widely confirms the stimulatory influence of meso-scale modifications on osteogenic differentiation, as described in Ogawa et al. and references therein [23]. Markers indicative of osteoblastic maturation showed a tendency toward enhancement on meso-structured surfaces compared with non-modified controls.

However, our data also indicate that the differentiation-promoting effects of meso-scale modifications are subtle and highly dependent on geometric specificity. The particular shape, depth, and substructural outcome of the meso-features appear to modulate the magnitude of the osteogenic response. This observation is especially relevant in the context of DLP-based ceramic additive manufacturing. In contrast to subtractive approaches, which can generate well-defined and mechanically robust meso-topographies, DLP printing is subject to material- and process-specific constraints.

The divergent transcriptional responses on 3Y and 5Y zirconia, particularly the strong and sustained BGLAP upregulation on 3Y-Mod-160 versus the more muted changes on 5Y surfaces, suggest that the same meso-scale geometry is filtered through material-specific micro- and nano-scale contexts. This view is consistent with recent work on hierarchically structured zirconia and the ‘missing middle’ concept in osseointegration [23,24,25,43]. Differences in phase composition, grain size and grain boundary density, surface energy, and sintering-induced residual stresses between 3Y-TZP and 5Y-PSZ are likely to alter protein adsorption profiles and integrin engagement, thereby modulating how osteoblasts sense and respond to identical meso-scale features [24,25,43]. A more pronounced promotion of terminal differentiation on 3Y may be beneficial where rapid stabilization of the bone–implant interface is desired, whereas the comparatively moderate response on 5Y could be compatible with clinical situations in which sustained proliferative capacity and matrix production are advantageous. These mechanistic interpretations remain speculative at this stage and will need to be corroborated by detailed surface physicochemical characterization and complementary protein-level and functional assays.

When benchmarked against recent high-impact work on hierarchically structured zirconia and titanium, our findings align with the general observation that meso-/micro-/nano-hybrid topographies can enhance osteoblast adhesion and differentiation without necessarily increasing proliferation. For example, Rezaei et al. and Saruta et al. reported comparable or slightly elevated early adhesion and ALPL/BGLAP expression on meso-nano hybrid zirconia relative to polished controls, while proliferation remained largely unchanged or modestly reduced. In the present DLP-printed 3Y zirconia, meso-scale Mod-160 structures achieved a similar qualitative pattern, with significantly increased adhesion and enhanced BGLAP expression at later time points, but unaltered growth kinetics. Quantitatively, the magnitude of BGLAP induction in our model appears somewhat lower than that reported for laser-generated grooves and spikes on zirconia and titanium, which may reflect both the subtler realized geometry after sintering and the absence of additional micro-/nano-scale texturing. These differences underscore that DLP-specific constraints currently limit the extent to which idealized meso-scale designs can be translated into robust, highly osteoinductive surfaces and point to the need for combined meso-/micro-structuring strategies in future work.

In ceramic DLP printing, the green body undergoes subsequent debinding and high-temperature sintering, processes that induce volumetric shrinkage, potential distortion of fine features, rounding of edges, and partial collapse of delicate structures. The final geometry may therefore deviate from the digital design, particularly in the meso-scale range where dimensional accuracy and structural stability are critical. These factors may attenuate or modify the intended biological cues of the meso-design, rendering the cellular response more subtle than anticipated from idealized geometries.

Consequently, the biological effect cannot be attributed solely to the nominal meso-scale geometry but must be understood as the result of a hierarchical surface state shaped by printing resolution, slurry composition, light scattering during photopolymerization, and sintering-induced grain growth. This complexity may partially explain why the differentiation-enhancing effects in our study, although confirmatory in direction, did not reach the magnitude reported for some laser-generated modifications on zirconia or titanium [50,51].

In DLP-printed zirconia, maintaining fracture toughness and fatigue resistance while introducing architecturally complex meso-features remains challenging, especially given sintering-induced shrinkage and potential defect formation. Our results also highlight the need for improved quantitative characterization of meso-scale topographies. Conventional roughness parameters such as Sa or Ra are insufficient to capture the spatial complexity and hierarchical organization of meso-features. The variability inherent to DLP printing further amplifies the necessity for validation of the final sintered geometry.

Considering our adhesion, proliferation and differentiation data together, a coherent biological pattern emerges: (i.) Proliferation was not impaired by meso-scale modifications. (ii.) Adhesion was significantly enhanced, and (iii.) differentiation showed a geometry-dependent enhancement.

Thus, our findings support the hypothesis that meso-scale topographies can help alleviate the classical proliferation–differentiation compromise of microrough implant surfaces.

Key limitations of our study include the use of a single immortalized cell line, indirect proliferation measurements, restricted differentiation markers, static culture conditions, and a comparatively low number of biological replicates for gene expression (*n* = 3), which limits statistical power and calls for confirmation of the observed trends in larger cohorts and at the protein and functional level. Moreover, DLP-specific manufacturing effects introduce hierarchical surface complexities that complicate direct attribution of biological responses to meso-scale geometry alone.

Although gene expression analysis provides valuable insights into osteogenic differentiation, mRNA levels do not necessarily correlate directly with protein expression or functional tissue formation. Post-transcriptional regulation, protein stability, and extracellular matrix mineralization processes are not captured by RT-qPCR analysis alone [52]. Therefore, conclusions regarding in vivo bone formation and osseointegration should be interpreted with caution.

Future studies should include additional experimental controls to strengthen both translational relevance and material characterization. In particular, human osteoblasts or mesenchymal stem cells would help determine whether the trends observed in hFOB 1.19 cells can be reproduced in more clinically representative cell populations. In addition, physicochemical surface characterization, such as contact angle measurements, surface energy analysis, protein adsorption assays, and the use of dynamic culture conditions, would provide a more comprehensive understanding of cell–material interactions. Another limitation of the present study is that meso-scale modified specimens were not included in cytotoxicity testing. Although the increased surface area and potential for residual processing artifacts may influence ion release, the prolonged sintering cycles required for these ceramics typically ensure high chemical stability and negligible cytotoxicity. Furthermore, the resins employed are already approved for clinical applications.

Finally, the present work is restricted to in vitro analysis of early cell–material interactions under static culture conditions and does not include in vivo assessment or mechanical evaluation of implant prototypes. As such, no direct conclusions can be drawn regarding primary stability, long-term fatigue behavior, or functional osseointegration. Future studies should therefore transfer the identified meso-scale designs to clinically relevant implant geometries and assess their biological and mechanical performance in appropriate animal models under load-bearing conditions.

## 5. Conclusions

This study provides evidence that meso-scale surface modifications on DLP-printed zirconia are biologically compatible, do not impair osteoblast proliferation, and can support osteogenic differentiation. At the same time, the differentiation-promoting effects appear geometry-dependent and sensitive to the specific manufacturing pathway and intrinsic properties of the materials used.

However, our findings also demonstrate that successful translation into clinically relevant zirconia implants requires precise control of printing and sintering processes, improved topographical characterization and individual cell biological validation. Thus, the study hypothesis was partially confirmed: meso-scale structures enhanced early osteoblast adhesion but did not significantly affect proliferation. Additionally, material-dependent effects on osteogenic differentiation were observed.

## Figures and Tables

**Table 1:** Primer and probes used for quantitative PCR.

Gene	Prmer/Probe	Sequence (5′ → 3′)	Label/Quencher	Size (bp)
ALPL	FH_ALPL	CTCTATCTTTGGTCTGGCCC	—	120
	RH_ALPL	AGACATTCTCTCGTTCACCG	—	
	PH_ALPL	[6FAM]GCAATGGGCCTGGCTACAAGGTGGT[OQA]	6FAM/OQA	
BGLAP	FH_BGLAP	TGTATCAATGGCTGGGAGC	—	123
	RH_BGLAP	GATAGGCCTCCTGAAAGCC	—	
	PH_BGLAP	[6FAM]TCCGGACTGTGACGAGTTGGCTGACCA[OQA]	6FAM/OQA	
COL1A1	FH_COL1A1	GGGAGAGACTGTTCTGTTCC	—	134
	RH_COL1A1	TGGTATAAAATGGGGAGCCG	—	
	PH_COL1A1	[6FAM]TTGATGTGTCACCGGGGCAACTGCC[OQA]	6FAM/OQA	
GAPDH	FH_GAPDH	GAAGGAAATGAATGGGCAGC	—	149
	RH_GAPDH	TCTAGGAAAAGCATCACCCG	—	
	PH_GAPDH	[6FAM]ACTAACCCTGCGCTCCTGCCTCGAT[OQA]	6FAM/OQA	
RPL27	FH_RPL27	ATCACCTAATGCCCACAAGG	—	126
	RH_RPL27	CTTCAAACTTGACCTTGGCC	—	
	PH_RPL27	[6FAM]TGCTCTTAAACGCAAGGCCCGACGGGA[OQA]	6FAM/OQA	
RUNX2	FH_RUNX2	CCAAGTAGCAAGGTTCAACG	—	108
	RH_RUNX2	TAGGTAGCTACTTGGGGAGG	—	
	PH_RUNX2	[6FAM]ATTTGTGGGCCGGAGTGGACGAGGCAA[OQA]	6FAM/OQA	
TBP	FH_TBP2	GGCTGTTTAACTTCGCTTCC	—	134
	RH_TBP2	TGTTCTGAATAGGCTGTGGG	—	
	PH_TBP2	[6FAM]TGCTCTTAAACGCAAGGCCCGACGGGA[OQA]	6FAM/OQA	

## Data Availability

Further data can be made available to interested parties on request to the corresponding author.

## Abbreviations

The following abbreviations are used in this manuscript:

3D	Three-dimensional
3Y-TZP	3 mol% yttria-stabilized tetragonal zirconia polycrystal
5Y-PSZ	5 mol% yttria partially stabilized zirconia
ALPL	Alkaline phosphatase, tissue-nonspecific isozyme
AM	Additive manufacturing
ATCC	American Type Culture Collection
BGLAP	Bone gamma-carboxyglutamate protein (osteocalcin)
bp	Base pairs
CAD	Computer-aided design
CAD/CAM	Computer-aided design/Computer-aided manufacturing
CC BY	Creative Commons Attribution License
cDNA	Complementary deoxyribonucleic acid
CO_2_	Carbon dioxide
COL1A1	Collagen type I alpha 1 chain
CT	Threshold cycle
ΔΔCT	Comparative threshold cycle method
DLP	Digital light processing
DMEM	Dulbecco’s Modified Eagle Medium
DNA	Deoxyribonucleic acid
ECM	Extracellular matrix
EDF	Extended depth of field
FBS	Fetal bovine serum
G418	Geneticin (antibiotic selection agent)
GAPDH	Glyceraldehyde 3-phosphate dehydrogenase
h	Hour(s)
hFOB 1.19	Human fetal osteoblast cell line 1.19
IU	International units
L929	Murine fibroblast cell line L929
mL	Milliliter
mM	Millimolar
mm	Millimeter
µm	Micrometer
MPa	Megapascal
mRNA	Messenger ribonucleic acid
M-MuLV RT	Moloney Murine Leukemia Virus Reverse Transcriptase
NA	Not applicable
OD	Optical density
PCR	Polymerase chain reaction
PSZ	Partially stabilized zirconia
qPCR	Quantitative polymerase chain reaction
RAI	Root-analogue implant
Ra	Arithmetical mean roughness
RNeasy	RNA isolation kit (Qiagen)
RNA	Ribonucleic acid
RPL27	Ribosomal protein L27
RT-PCR	Reverse transcription polymerase chain reaction
RUNX2	Runt-related transcription factor 2
Sa	Arithmetical mean height (surface roughness parameter)
SC	Sintering shrinkage coefficient (context-dependent)
SD	Standard deviation
SEM	Scanning electron microscopy
TBP	TATA-binding protein
TCP	Tissue culture plastic
TZP	Tetragonal zirconia polycrystal
*v*/*v*	Volume per volume
ZrO_2_	Zirconium dioxide
